# Differences in perceptions and fast food eating behaviours between Indians living in high- and low-income neighbourhoods of Chandigarh, India

**DOI:** 10.1186/1475-2891-12-4

**Published:** 2013-01-07

**Authors:** Christopher Robert Aloia, Danijela Gasevic, Salim Yusuf, Koon Teo, Arun Chockalingam, Binod Kumar Patro, Rajesh Kumar, Scott Alexander Lear

**Affiliations:** 1 Faculty of Health Sciences, Simon Fraser University, Burnaby, BC, Canada; 2 Department of Biomedical Physiology and Kinesiology, Simon Fraser University, Vancouver, BC, Canada; 3 Faculty of Medicine, McMaster University, Hamilton, Ontario, Canada; 4 Office of Global Health, National Heart Lung and Blood Institute, Bethesda, MD, USA; 5 Community Medicine, Post Graduate Institute of Medical Education and Research, Chandigarh, India; 6 Division of Cardiology, Providence Health Care, Vancouver, Canada

**Keywords:** Fast food, Neighbourhood income, Street food, India, South Asian

## Abstract

**Background:**

Increased density of fast food restaurants is associated with increased prevalence of obesity in developed countries. However, less is known about this relationship in developing countries undergoing rapid urbanization and how differences in neighbourhood income affect the patronage of fast food outlets. The purpose of the study is to explore the differences in fast food preferences, perceptions, and patronage between Indians living in high- and low-income neighbourhoods.

**Methods:**

This cross-sectional study recruited 204 men and women (35 to 65 years in age) from high- and low-income neighbourhoods who completed a questionnaire on fast food consumption. The questionnaire asked participants to define fast food and to provide reasons for and frequency of visits to fast food restaurants. The differences were analyzed using Chi square and t-tests for categorical and continuous variables, respectively.

**Results:**

Participants from a high-income neighbourhood were more likely to perceive Western -style fast food as fast food, while people from the low-income neighbourhood were more likely to identify food sold by street vendors as fast food (p <0.001). Furthermore, compared to participants from the high-income neighbourhood, people from the low-income neighbourhood were more likely to report buying food from street vendors while less likely to dine out at both fast food and non-fast food restaurants (p<0.001). Although the high-income neighbourhood group was more likely to report enjoying eating at fast food restaurants than their low-income neighbourhood counterparts, there were no significant differences in the reasons for visiting fast food restaurants (convenience, price, social enjoyment, and quality of meals) between the two groups. Both groups preferred home cooked over restaurant meals, and they recognized that home cooked food was healthier.

**Conclusions:**

Overall, consumption of fast food was low. People from a high-income neighbourhood dined out more frequently and were more likely to perceive Western-style food as fast food compared to their counterparts from the low-income neighbourhood.

## Background

In the past few decades, fast food (food prepared in a restaurant with limited service staff and from which the majority of meals are consumed off premises) has been implicated as one of the contributors to increased population rates of obesity [1-4]. The growth of the fast-food industry has led to an increased consumption of food prepared away from home that is high in total and saturated fat, as well as sodium, but low in dietary fibre, calcium, and iron [5]. Prospective data from Western countries have shown that there is a positive association between frequency of fast food restaurant use and weight gain [4,6,7]. Hence, as Western fast food companies are expanding in developing countries such as India [8], there is a considerable concern that such countries are in danger of succumbing to the same obesity trends as in the Western countries [9].

In recent years, there has been a marked increase in the rates of obesity in countries such as India that has been attributed to unhealthy lifestyle practices associated with the introduction of Western-style fast foods that are higher in fat and refined carbohydrates [10,11]. Yadav and Krishanan found that the prevalence of central obesity in North India increased with the level of urbanization in both men and women by 8.7% and 34.5%, respectively [11]. This is important, as central obesity is associated with less desirable cardiovascular risk profile. Indeed, it has been shown that, at comparable BMI and age, South Asians were at increased cardiovascular risk compared to their Caucasian counterparts [12-14]. Given that fast food is implicated as one of contributors to the increase in obesity rates [2], understanding the perceptions of fast food and reasons for eating at fast food restaurants in India will help inform health promotion strategies.

Research on people’s perceptions about fast food in India is limited. Results of a study that surveyed Indian adults in their 20s showed that, although they preferred home cooking over fast food, their main reasons for visiting fast food establishments were going there for fun, change of scenery, and socializing [8]. In another recent study that surveyed 106 South Asians from Bangladesh, researchers revealed that consumers are most likely to buy lunch or midday snack at a Western-style fast food restaurant and consider costs, mood of the restaurant, availability of various types of food, convenience, and location of the restaurant in their decision [15]. While these studies did investigate South Asian people’s preferences regarding fast food, they did not explore whether those differ between people living in neighbourhoods of differing income levels. This is important given that neighbourhood income was found to be associated with fast food density and obesity in developed countries [6,16,17]. Consequently, the main objectives of this study were to explore the differences in fast food consumption between Indians adults (35 to 65 years of age) living in high- and low-income neighbourhoods in India and to explore if the difference in neighbourhood income affects their patronage of fast food restaurants and their perception of fast food itself.

## Methods

This cross-sectional study was conducted as part of the Prospective Urban Rural Epidemiology (PURE) study, which examines the relationship of societal influences on human health related behaviours, cardiovascular risk factors, and incidence of chronic, non-communicable diseases [18]. This sub-study is based on a sample of convenience recruited from June to August 2009 at the Post-Graduate Institute of Medical Education and Research (PGIMER), Chandigarh, India. All individuals between ages 35 and 65 recruited for the PURE study within the above mentioned period agreed to fill out an additional questionnaire on fast food behaviour and participate in this study. In total, 204 men and women participated in this study, out of which 103 individuals were from a high-income neighbourhood, while 101 men and women came from a low-income neighbourhood. Neighbourhood income was determined by average house size in the neighbourhood. House size is measured objectively via a census. The houses measuring 100 square yards and less are considered low-income, while the houses measuring more than 250 square yards are considered high-income. Neighbourhood where houses measuring 100 square yards and less were predominant was defined as low-income neighbourhood, while neighbourhood where houses bigger than 250 yards were predominant was considered high-income neighbourhood. Individuals from both neighbourhoods were approached in the same manner reflecting the internal validity of the study. All participants were assessed for smoking behaviour, educational attainment, household income, marital status, and religion as part of the PURE study. Additionally, each participant filled out a questionnaire in order to be assessed for fast food behaviour. Fast food was defined as food with distinctly “Western” characteristics, such as sandwiches, take-out pizza, deep fried chicken, or burgers. Researchers defined fast food establishments as restaurants that either had foods prepared or took little time to prepare, with limited service staff and the majority of business derived from take-out sales [11]. Fast food restaurants included McDonald’s, Pizza Hut, and Kentucky Fried Chicken, which were included on the questionnaire because they were the most common Western-style fast food restaurants in Chandigarh [19-21]. All participants provided informed consent to the study. The Simon Fraser University Office of Research Ethics Board approved this study*.*

### Fast food behaviour

Fast food behaviour was measured using a survey instrument specifically developed to explore the differences in fast food perception and consumption between individuals from high- and low-income neighbourhoods in Chandigarh, India. The face validity of the survey instrument was assessed through an in-person interview, which was conducted with an individual from Chandigarh to test its cultural appropriateness as well as with individuals in Canada who emigrated from the region in and around Chandigarh, India. However, no formal test of validity was performed. The questionnaire asked participants how they define fast food, about the frequency of fast food dining and buying snacks/desserts outside the home, whether or not they enjoyed eating at fast food restaurants, and the reasons for eating at fast food restaurants. Furthermore, a question on buying food from a local street vendor was included in the questionnaire, given that in developing countries such as India, buying food from a street vendor is an affordable and convenient meal option when eating outside the home. In addition, questions were asked about the frequency of eating at specific fast food restaurants and traditional restaurants not considered fast food. It is important to note that many of the participants rarely patronized fast food restaurants, so the questions pertaining to frequency of eating outside the home, originally given in days per week, are presented as the average number of times per year.

Furthermore, dining out for breakfast and lunch was very rare among participants from both low- and high-income neighbourhood groups. There were only 9 people (out of 204) that reported dining out for breakfast (3 people from low-income and 6 people from high-income neighbourhoods). Similarly, 30 people reported dining out for lunch (15 in each neighbourhood group). Consequently, in order to increase power we collapsed breakfast, lunch, and dinner meals and explored the neighbourhood differences in dining out for breakfast, lunch, and dinner. Participants were further asked about distance from home to fast food restaurants. The survey instrument also asked participants’ perceptions on which they considered healthier: food cooked at home, or food prepared outside the home at non-fast food or fast food establishments. Two experienced interviewers fluent in the three most common local languages (Punjabi, Hindi, and Urdu) administered the questionnaire by interview. It is important to note that although one of the initial questions asked participants what closely matched their definition of fast food, the interviewers then explained to all participants that the remaining questions asking about fast food pertained specifically to fast food with distinctly “Western-style” characteristics, such as sandwiches, take-out pizza, deep fried chicken, and burgers. Having one of the authors (CRA) present at all interviews ensured that all questions were uniformly understood by participants. The survey instrument is available upon request.

### Statistical analysis

Variables are presented as counts and percentages if categorical or as means and standard deviations if continuous. Non-normally distributed continuous variables are presented as geometric means (95% CI). The differences in tested parameters between participants from high- and low-income neighbourhoods were explored using an independent t-test (normally distributed continuous variables), Mann U Whitney test (non-normally distributed variables), or a Chi square (χ^2^) test (categorical variables). Analysis of the question that probes people’s reasons for going to fast food restaurants was performed only on participants who reported eating at fast food restaurants. A *p*-value of 0.05 and less was considered statistically significant. All analyses were performed using SPSS version 17.0, SPSS Inc., Chicago, IL.

## Results

Participants from the high-income neighbourhood were younger and smoked less than participants from the low-income neighbourhood (Table 1). The high-income neighbourhood comprised Hindus (69.9%), Sikhs (28.2%) and Jains (1.9%). Participants living in the low-income neighbourhood were predominantly of Hindu religious background (88.1%) with fewer than half as many Sikhs than in the high-income neighbourhood (11.9% vs. 28.2%). The high-income neighbourhood had a greater prevalence of illiteracy (24.2% vs. 3.0%) and fewer people with secondary level of formal education (16.5% vs. 37.6%) than the low-income neighbourhood. Percentage of people with higher than secondary level of formal education was similar in high- and low-income neighbourhoods (44.7% vs. 48.5%). There was no significant difference in household income between individuals living in high- and low-income neighbourhoods (p>0.05).

**Table 1:** Socio-demographic characteristics of study participants

	**High-Income Neighbourhood**	**Low-Income Neighbourhood**	
	**n = 103**	**n = 101**	
Males	50 (49.5%)	51 (49.5%)	1.00
Age	51.3 ± 8.9	49.9 ± 7.4	0.002
Married	99 (96.1%)	100 (99.0%)	0.181
Current smoking	1(1.0%)	10 (9.9%)	0.005
Ethnicity			0.004
Hindu	72 (69.9%)	89 (88.1%)	
Sikh	29 (28.2%)	12 (11.9%)	
Jain	2 (1.9%)	0 (0.0%)	
Formal education			< 0.001
Illiterate	25 (24.2%)	3 (3.0%)	
Primary	15 (14.6%)	11 (10.9%)	
Secondary	17 (16.5%)	38 (37.6%)	
Higher than secondary	46 (44.7%)	49 (48.5%)	
Household income quartiles*			0.461
Rs. 1000 – 6000	33 (32.1%)	33 (32.7%)	
Rs. 6001 – 10000	26 (25.2%)	33 (32.7%)	
Rs. 10001 – 20000	26 (25.2%)	24 (23.8%)	
20001 – 35000	18 (17.5%)	11 (10.8%)	

Categorical variables presented as n (%); continuous variables presented as mean ± SD; Differences in age tested by independent t test and in the rest of variables using Chi-square test.

* Monthly personal income 100 Indian rupees equals $2 USD; exchange rate 0.02013 [49.6736] on May 1st, 2009 (date of participant recruitment), Bank of Canada http://www.bankofcanada.ca/rates/exchange/10-year-converter/.

Perception of fast food was measured by asking the question, “Which most closely matches your definition of ‘fast food’”? Options participants could choose from were “food sold by street vendors, such as bhel puri or samosas”, “food served at restaurants such as McDonald’s or Kentucky Fried Chicken”, or “no answer”. Participants from a high-income neighbourhood were more likely to report fast food as food sold at chain restaurants. In contrast, individuals from a low-income neighbourhood were four times more likely to report fast food as food sold by street vendors compared to their counterparts from a high-income neighbourhood (Table 2).

**Table 2:** Differences in perceptions regarding fast food between individuals from high- and a low-income neighbourhoods

		**High-income neighbourhood**	**Low-income neighbourhood**	*p *value*
		**n = 103**	**n = 101**	
Which most closely matches your definition of fast food?	Sold by street vendors	8 (7.8%)	33 (32.7%)	< 0.001
	Sold at chain restaurants	88 (85.3%)	45 (44.6%)	
	No answer	7 (6.9)	23 (22.7%)	
Do you enjoy eating at fast food restaurants?	Yes	31 (30.0%)	10 (9.9%)	0.002
	No	36 (35.0%)	41 (40.6%)	
	Do not eat	36 (35.0%)	50 (49.5%)	
How close is the nearest fast food restaurant to your home	< 0.5 km	13 (12.6%)	9 (8.9%)	0.088
	(0.5-1.5) km	31 (30.1%)	34 (33.7%)	
	(1.5-3.5) km	36 (35.0%)	47 (46.5%)	
	> 3.5 km	23 (22.3%)	11 (10.9%)	
If a fast food restaurant were closer to your home would you go out to eat there more?	Yes	2 (1.9%)	5 (5.0%)	0.117
	No	98 (95.2%)	96 (95.0%)	
	Maybe	3 (2.9%)	0 (0.0%)	
Which do you think is healthier?	Food you eat at home	102 (99.0%)	101 (100.0%)	0.549
	Restaurant food	1 (1.0%)	0 (0.0%)	
Which do you prefer?	Home cooking	102 (99.0%)	100 (99.0%)	0.368
	Restaurant dining	1 (1.0%)	1 (1.0%)	

*results of Chi-square test.

There was a significant association between neighbourhood income and whether or not people enjoyed eating at fast food restaurants (p = 0.002) (Table 2). Participants from the high-income neighbourhood were more likely to report enjoying eating at fast food restaurants compared to people from a low-income neighbourhood (30.1% vs. 9.9%). However, a substantial percentage of participants from both high-income (35%) and low-income (49.5%) neighbourhoods reported not eating fast food at Western-style fast food restaurants. In addition, about 95% of participants from both high- and low-income neighbourhood groups reported that having a fast food restaurant closer to their home would not make them eat at a fast food restaurant more often. Also, almost all participants reported that they prefer home cooking to and believe it is healthier than restaurant food (Table 2).

All participants from high- and low-income neighbourhoods were assessed for differences in frequencies of eating outside the home. Table 3 shows that frequency of eating outside of the home was low for both residents of high-income and low-income neighbourhoods. However, compared to people from the low-income neighbourhood, individuals from the high-income neighbourhood reported more frequent consumption of breakfast, lunch, and dinner outside the home (5.9 vs. 2.3 times per year; p < 0.001). Furthermore, participants from the high-income neighbourhood reported more frequent visits to fast food restaurants such as Pizza Hut, McDonald’s, and Kentucky Fried Chicken (3.5 vs. 1.4 times per year; p < 0.001), and they dined more often at traditional, non-fast food restaurants compared to their counterparts living in the low-income neighbourhood (6.0 vs. 2.7 times per year; p<0.001). In contrast, people from the low-income neighbourhood were more likely to report buying food from street vendors compared to those in the high-income group (6.5 vs. 2.7 times per year; p<0.001). Participants from the high-income neighbourhood consumed snacks or desserts outside the home more than participants living in the low-income neighbourhood, although this did not reach statistical significance (p = 0.065).

**Table 3:** Frequency of dining out per year for individuals from high- and low-income neighbourhoods

**Eating out for/at:**	**High income**	**Low-income**	*p *value*
	**n = 103**	**n = 101**	
Snacks or desserts	13.5 (10.3, 17.7)	10.4 (8.0, 13.7)	0.065
Breakfast, lunch and dinner	5.9 (4.3, 8.0)	2.3 (1.7, 3.2)	< 0.001
Street vendor	2.7 (2.0, 3.5)	6.5 (4.9, 8.5)	< 0.001
Fast food establishments	3.5 (2.7, 4.5)	1.4 (1.1, 1.9)	< 0.001
Traditional restaurant (not fast food)	6.0 (4.6, 7.8)	2.7 (2.0, 3.5)	< 0.001

Variables presented as geometric means (95% CI); *results of Mann U Whitney test.

Subsequent questions (Table 4) probed people’s reasons for eating at fast food restaurants. These questions were directed only to participants who reported eating at fast food restaurants (64 and 43 residents of high- and low-income neighbourhoods, respectively). There was no difference between people from high- and low-income neighbourhoods regarding reasons for eating at fast food restaurants, including convenience, price, social enjoyment, and quality of meals. In addition, neither group of participants reported price as a reason for going to fast food restaurants nor did they report that they ate too much fast food.

**Table 4:** Reasons for going to fast food establishments of those participants who have patronized them

	**High-income neighbourhood**	**Low-income neighbourhood**	*p *value*
	**n = 64**	**n = 43**	
Do you go to fast food restaurant because of:			
Convenience	26 (40.6%)	15 (34.9%)	0.357
Price	0 (0.0%)	0 (0.0%)	N/A
Social enjoyment	53 (82.8%)	39 (90.7%)	0.249
Quality of meals	4 (6.2%)	2 (4.7%)	0.725
Do you think you eat too much fast food?	0 (0.0%)	1 (2.3%)	0.220

*results of Chi-square test.

## Discussion

Through our investigation we sought to explore the differences between Indian individuals living in a high- and those living in a low-income neighbourhood in Chandigarh, India, regarding their patronage of fast food restaurants, perception of fast food, and fast food consumption. The results of our study indicate that people from a high-income neighbourhood dined out more frequently and were more likely to perceive Western-style food as fast food, while people from the low-income neighbourhood were more likely to identify food sold by street vendors as fast food. Based on the questions asked, there were no significant differences in the reasons for visiting fast food restaurants between the two groups, such as convenience and price, and both groups were more in favour of home-cooked over restaurant meals.

According to the Indian Fast Food Market Analysis report, India’s fast food market is growing quickly, at a rate of 30-35% per year [22]. It has been suggested that rapid urbanization, swift economic growth, and increase in average income in India will lead to an increase in consumerism [23]. Our study found that individuals from the high-income neighbourhood were more likely to eat at fast food restaurants compared to their counterparts from the low-income neighbourhood. Furthermore, a large minority of our study participants (23%) were not familiar with what fast food is, while another 33% cited local street vendors as fast food providers, as opposed to Western-style fast food restaurants. Some classic examples of Indian street food include samosa, a deep-fried pocket of spiced mixture of potato and peas inside a dough made primarily of chickpea flour; channa batura, which is spicy chickpeas served with a deep-fried, puffy dough; and pav bhaji, a spicy, grill-fried puree of tomatoes, carrots, and peppers served with white-bread rolls. It is interesting to note that most street vendors also use their own recipes and make everything by hand, using a variety of oils and ingredients, so the nutritional content of the same type of food may differ from vendor to vendor. This greater consumption of Western-style fast food in the higher income group is in contrast to findings in studies in the West that indicate greater consumption among low income individuals [24]. This may be the result of strategic marketing by Western fast food companies defined as “glocalisation,” which describes a multinational company that tailors products and marketing specifically to local cultural tastes [25]. In addition, when a fast food company enters a foreign market they usually target the middle class and higher [26,27]. Consequently, given that entry of Western-style fast food branded products to India has been facilitated for the more affluent consumers, eating Western-style fast food on a regular basis is still financially out of reach for low-income people [28]. However, in our study, none of the participants cited price as impacting their decision to eat at these establishments. Furthermore, although the frequency of eating at Western-style fast food restaurants was higher among individuals in the high-income neighbourhood compared to their counterparts from the low-income neighbourhood, the frequency of eating at fast food restaurants was generally low among people from both neighbourhoods.

The Chandigarh Healthy Heart Action Project (CHHAP) reported that more people aged 15–24 years old living in an urban area (72%) preferred Western-style fast food than people of the same age in rural areas (37%) [29]. In our study, we reported that 30% of the high-income group enjoyed eating fast food compared to 10% of the low-income. Preference levels for fast food appear to be quite lower than the CHHAP findings. This difference may be due to the fact that we sampled people from a low-income neighbourhood where these participants indicated eating less at Western fast food restaurants. Another explanation for this difference could be due to our study population being older than that in the CHHAP study. Studies in the US generally indicate that fast food consumption is greater among younger people [30].

We also found that in both high- and low-income study participants, home cooked food was preferred over food bought in fast food establishments and that nearly all participants indicated that home cooked foods are healthier than fast food. This is consistent with the results of an earlier study, which indicated that younger Indian fast food consumers prefer home cooked meals to fast food [8]. One of the reasons could be that in the West, fast food more closely resembles food typically served throughout the culture, whereas in India, Western-style fast food and Indian home cooked food are quite different. Although restaurants have tried to make and adapt food to local tastes, Western-style fast food is still categorically different than traditional fare [26]. Nevertheless, high-income participants reported eating at Western-style fast food restaurants more often, but there was no difference between the two groups with respect to the reasons for eating fast food. It should also be noted we found that eating for social enjoyment was the highest cited reason, followed by convenience, whereas Farhana and Islam found that brand loyalty and quality were the main reasons for consuming fast food in people living in Dhaka, Bangladesh, with a mean age of 25 [15]. Goyal and Singh, who also found quality and value as the main reason why people patronize fast food restaurants, substantiate this point [8]. Interestingly, we also asked if the quality of meals were a reason for going to fast food restaurants and the response was over 90% negative. Again, age and regional differences might provide a reason for the discrepancy of the findings.

Given the rapid expansion of fast food industry in India, and in the light of the negative impact of fast food on health [1], it is important to understand how Indians perceive fast food and the reasons they patronize fast food businesses. Given that fast-food consumption has been associated with increases in body weight and insulin resistance, it is further implicated in the development of Type 2 diabetes [4]. In general, people of South Asian background are at greater risk for CVD and diabetes compared to other ethnic groups [12,13,31]. While most participants reported they would not eat fast food even if it were closer to them, the rapid expansion of fast food establishments will increase availability and likely reduce cost of fast food. These factors have been found to increase fast food consumption in other countries undergoing rapid economic growth [3].

### Limitations

This study employed a cross-sectional design, so no conclusions can be made about the causal relationships between the variables. Moreover, only individuals between ages of 35 and 65 years were surveyed. Younger generations are typically the higher consumers of fast food, which may have obstructed a positive finding of higher levels of fast food patronage in a younger cohort. However, surveying older consumers is as important as surveying younger ones, as factors influencing choice of fast food establishments as well as reasons for visiting and eating at fast food outlets might be different for individuals of various age groups. The higher rate of illiteracy in the defined high-income neighbourhood was unexpected. Factors,such as self-reporting bias can play a role. Furthermore, according to Indian Census, 47% of the population rents a place [32]. Consequently, it is possible that people living in the house are not necessarily always the owners of the house but rather people who are renting the place and are from various educational and income brackets. Furthermore, in our study, we did not notice a statistically significant difference in individual income between people living in high- and low-income neighbourhoods. It has been established that people tend to deny socially undesirable traits or qualities and report more socially desirable ones instead [33]. Furthermore, others reported that high-income individuals may be reluctant to disclose their income [34]. Consequently, there is a possibility that income was misreported within the sample, with people from the high-income neighbourhood under-reporting and those from the low-income neighbourhood over-reporting personal income. Furthermore, as noted above, people occupying high-income neighbourhood houses may not necessarily be house owners but rather the tenants renting a place and coming from various income brackets. Moreover, study participants surveyed come only from two neighbourhoods in Chandigarh, so the results cannot be generalized to the whole city of Chandigarh or other cities/regions of India. In addition, given that reported consumption of fast food was low in our study sample, we did not have enough power to explore whether socio-demographic characteristics modify the relationship between neighbourhood income and fast food consumption.

## Conclusions

Indians from a high-income neighbourhood were more familiar with fast food as it is defined in the West, and they dined at fast food restaurants more frequently. Furthermore, they were more likely to report that they enjoy eating at Western-style fast food restaurants compared to their low-income neighbourhood counterparts. On the other hand, Indians living in a low-income neighbourhood were more likely to buy and report food sold by street vendors as fast food. Overall, consumption of fast food was low among a population of Indians from Chandigarh. Future studies should investigate whether rapid expansion of fast food establishments will lead to an increase in fast food consumption among Indians what was noted in other countries undergoing rapid economic growth.

## Competing interests

The authors declare no competing interests in the writing and researching of the information in this paper.

## Authors’ contributions

CRA conceived of the study, constructed the questionnaire, carried out the data analysis, and drafted the manuscript. DG carried out the data analysis and drafted the manuscript. SY, KT, SAL, and AC participated in the design of the study and construction of the questionnaire. RK and BKP participated in study design and coordination. All authors read, reviewed, and approved the final manuscript.
